# Cost-effectiveness analysis of text messaging to support health advice for smoking cessation

**DOI:** 10.1186/s12962-021-00262-y

**Published:** 2021-02-15

**Authors:** Raquel Cobos-Campos, Javier Mar, Antxon Apiñaniz, Arantza Sáez de Lafuente, Naiara Parraza, Felipe Aizpuru, Gorka Orive

**Affiliations:** 1 Bioaraba Health Research Institute, Epidemiology and Public Health Research group, Vitoria-Gasteiz, Spain; 2 Osakidetza Basque Health Service, Primary Care Research Unit of Gipuzkoa, Alto Deba Hospital, Arrasate-Mondragón, Spain; 3 Health Services Research on Chronic Patients Network (REDISSEC), Bilbao, Spain; 4 Biodonostia Health Research Institute, San Sebastián, Spain; 5 Osakidetza Basque Health Service, Lakuabizkarra Health Centre, Vitoria-Gasteiz, Spain; 6 Preventive Medicine and Public Health Department, University of Basque Country UPV/EHU, Vitoria-Gasteiz, Spain; 7 Osakidetza Basque Health Service, Subdirectorate of Health Care, Vitoria-Gasteiz, Spain; 8 Health Services Research on Chronic Patients Network (REDISSEC), Vitoria-Gasteiz, Spain; 9 NanoBioCel Group, Laboratory of Pharmaceutics, School of Pharmacy, University of the Basque Country UPV/EHU, Vitoria-Gasteiz, Spain; 10 Bioaraba Health Research Institute, Nanobiocel Research group, Vitoria-Gasteiz, Spain; 11 University Institute for Regenerative Medicine and Oral Implantology - UIRMI (UPV/EHU-Fundación Eduardo Anitua), Vitoria-Gasteiz, Spain; 12 Singapore Eye Research Institute, Singapore, Singapore; 13 CIBER Bioengineering, Biomaterials and Nanomedicine (CIBERBBN), Institute of Health Carlos III, Madrid, Spain

**Keywords:** Smoking cessation, MeSH unique ID: D016540, Costs and cost analysis, MeSH unique ID: D003365, Cost of illness, MeSH unique ID: D017281, Quality-adjusted life years, MeSH unique ID: D019057, Text messaging, MeSH unique ID: D060145

## Abstract

**Background:**

Smoking in one of the most serious public health problems. It is well known that it constitutes a major risk factor for chronic diseases and the leading cause of preventable death worldwide. Due to high prevalence of smokers, new cost-effective strategies seeking to increase smoking cessation rates are needed.

**Methods:**

We performed a Markov model-based cost-effectiveness analysis comparing two treatments: health advice provided by general practitioners and nurses in primary care, and health advice reinforced by sending motivational text messages to smokers’ mobile phones. A Markov model was used in which smokers transitioned between three mutually exclusive health states (smoker, former smoker and dead) after 6-month cycles. We calculated the cost-effectiveness ratio associated with the sending of motivational messages. Health care and society perspectives (separately) was adopted. Costs taken into account were direct health care costs and direct health care cost and costs for lost productivity, respectively. Additionally, deterministic sensitivity analysis was performed modifying the probability of smoking cessation with each option.

**Results:**

Sending of text messages as a tool to support health advice was found to be cost-effective as it was associated with increases in costs of €7.4 and €1,327 per QALY gained (ICUR) for men and women respectively from a healthcare perspective, significantly far from the published cost-effectiveness threshold. From a societal perspective, the combined programmed was dominant.

**Conclusions:**

Sending text messages is a cost-effective approach. These findings support the implantation of the combined program across primary care health centres.

## Background

Smoking in one of the most serious public health problems [1]. It is well known that it constitutes a major risk factor for chronic diseases and the leading cause of preventable death worldwide [2]. In Spain, according to the 12th Survey on Alcohol and other Drugs (EDADES), 34% of people between 15 and 64 years old admit to smoking on a daily basis in the past month, which represents an increased rate compared with rates (30 and 31%) from previous surveys (2011, 2013 and 2015) [3]. Smoking is associated with higher healthcare costs, with an estimation of €864.64/year in smokers versus €474.71/year in non-smokers according to a study by the Spanish Society of Pulmonology and Thoracic Surgery [4].

Currently, there are various different treatments for smoking cessation, including more or less intensive interventions based on motivational advice, pharmacological therapy and group-based programs, with variable success rates depending on the therapy used [5]. On the other hand, since every year more than 70% of the population goes to primary care room, and they are attended an average of 6–7 times, this privileged situation allows for repeated interventions in which the smoker is more receptive to motivational advice because of the trust they have with their doctor and/or nurse [6]. For this reason we must take advantage of the opportunity provided by primary care to provide this type of intervention from there. In Spain, several studies have been conducted to evaluate tobacco cessation interventions at this level with satisfactory results [7, 8]. Interestingly, some smokers decide to quit smoking without any support, with success rates varying from 3 to 8% after 6 months [9, 10].

The provision of health advice is considered one of the most cost-effective interventions for smoking cessation [11]. However, changes stimulated by such advice do not last over time [12] and hence, there is a need to establish approaches for reinforcement, including the use of information and communication technologies, and specifically m-health (health through mobile technologies) for which there is evidence in smoking cessation [13–16]. Mobile phone based interventions are more effective that routine clinical practice, according a meta-analysis of six clinical trials carried out by Whittaker et al. [13] 1.83 (95% CI 1.54–2.19), being results similar to those reported by other research groups [14–16].

We carried out a randomized clinical trial to assess the effectiveness of a combined program SMSalud® that included sending motivational messages by mobile phone to smoker people who sought help from primary health professionals, finding 16.25% of abstinences rates at 12 months versus 5.6% of standard care group) [16]. Further, with the deployment of mobile networks in the nineteen-eighties, the use of mobile phones has grown exponentially. The International Telecommunications Union estimated that by the end of 2015 there would be 7 billion mobile phones across the world, corresponding to a penetrance of 97% [17], and their increasingly widespread use makes these devices ever more useful tools in healthcare.

Assuming that mobile phones are useful tools in healthcare, it seems reasonable to explore strategies focused on using mobile technology to improve smoking cessation. Although the combined program SMSalud® has shown to be effective as a tool to reinforce health advice provide in primary care health professionals, and the results of Guerriero et al. [18] suggest that it would be a cost-effective tool, there is a need for long-term specific economic assessment prior its implementation in the primary care setting. Taking all this into consideration, the main objective of the present article was to assess whether the use of text messages as a support tool for health advice is a cost-effective strategy in smoking cessation programs in primary care.

## Methods

### Model

We performed a Markov model based cost-effectiveness analysis to calculate the incremental cost-effectiveness ratio (ICUR), which is a measure that compares differences in costs and differences in effectiveness between the options considered.

**Fig. 1:**
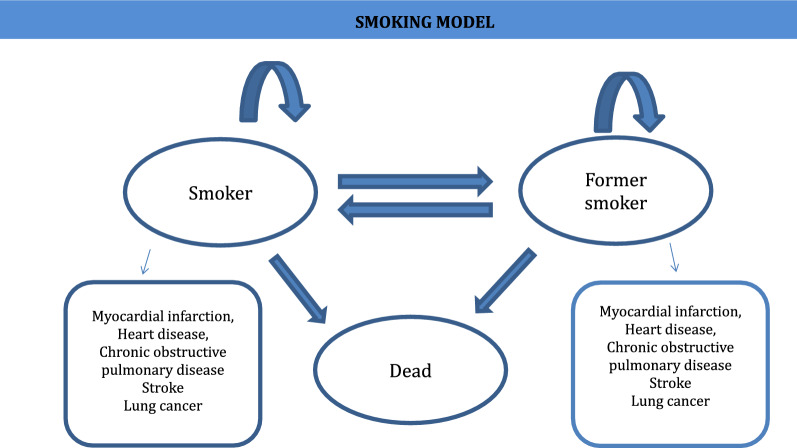
.

To estimate the costs and clinical outcomes from the start of the intervention until patient death, we used a Markov model that has been used previously in economic assessments [18–20]. This Markov model consists of three mutually exclusive health states (smoker, former smoker and dead), to simulate the process of smoking cessation in a hypothetical cohort of 1000 smokers aged 16 years old or above. Specifically, we opted for a model with cycles of 6 months, in which people transitioned between the three health states, with transition probabilities differing as a function of time, age and sex (Fig. 1). All people started the model in the smoker state, and in the first cycle, could then stop smoking, continue smoking or die. From the second cycle onwards, participants could continue to not smoke, continue to smoke, start smoking again or die. Additionally, both smokers and former smokers could develop smoking-related diseases (myocardial infarction, stroke, heart disease, chronic obstructive pulmonary disease or lung cancer), with different probabilities as a function of age and gender. For calculating the incremental cost associated with the reinforcement provided through mobile text messaging, the time horizon was set to be the patient´s entire life. This time frame allowed us to include both the health impact and all the costs associated with smoking over a patient´s life and thus explore the reduction in costs and improvement of quality of life due to the use of reinforcing text messaging.

As well as having implications for the health of smokers, it also has a major impact on public finances in both the short and long term, since tobacco-related diseases are chronic diseases that cause high health expenditure. The probability of suffering from these diseases increases with the age of the patient, so it is necessary to include the time horizon of the patient's entire life.

A discount rate of 3% was used for updating future costs and health benefits. This discount rate is widely used in long-term cost-effectiveness studies [21–23]. The analysis was conducted from a health system perspective including all the costs related to the intervention provided and costs related to the five SRD and from a societal perspective including in addition to all the above, the costs due to loss of productivity caused by sick leave associated with the five diseases most strongly associated with smoking.

### Alternatives

The study was based on the aforementioned clinical trial in which we assessed the effectiveness of the combined program, SMSalud®, comparing it with health advice alone [16].

We compared the following two treatment options: (a) Usual clinical practice carried out in primary care health centers- health advice provided by general practitioners and nurses responsible for smoking cessation management (verbal and written information on the benefits of not smoking and recommended changes in eating habits) [16], and (b) the same health advice complemented by the sending of motivational and supportive messages to the patient´s mobile phone during the 6 months the program lasted (combined program). Health advice was provided repeatedly (at 7 days, 4, 12 weeks and at 6 months since the quitting day). In both cases, the treatment lasted 6 months. Smokers who participated in the previous clinical trial had a mean age of 45 years old (SD 9.1). The 55.94% of participants were men and they were included in the study if they obtained a score of > 4 on the Richmond test and if they didn’t have depression. The percentage of patients who quitted smoke at 6 months was 24% (95% CI 31–17.72%) and 12% (CI 95% 6.686–16.88%) in the intervention and control group respectively. At 12 months smoking cessation rates were 16.25% (95% CI 10.53–21.97) and 5.6% (95% CI 2.04–9.16) [16].

Participants on the combined program received two automatically-generated text messages a day (one in the morning and one in the evening) for the first 5 weeks and three messages a week from weeks 6 to 26. At 26 week, the program finished. The messages were motivational in intent, to encourage people in their efforts to stop smoking, and also provided information about the health-related risks of smoking. Participants could also request supportive messages from the system in moments of crisis or anxiety. For this, they had to send a message free of charge with the word “anxiety” or “relapse” to a given phone number [16].

### Probabilities

The probabilities of smoking cessation and relapse used in this cost-effectiveness study are taken from our previous clinical trial [16] in 320 smokers. In this clinical trial, 24% (95% CI 17.72–31%) of people who received reinforcement messages stopped smoking after 6 months, compared to 12% (95% CI 6.86–16.88%) of people assigned to health advice alone, not founding statistically significant differences in success rates between men and women. The rates of relapse were 33% and 53% in the groups receiving health advice plus reinforcement messages and health advice alone respectively (Table 1).

**Table 1:** Data entered into the model

Inputs			Source
Mortality	By smoking status (smoker, former smoker)		27
Prevalence of smoking-related diseases	Myocardial infarction		19
	Heart disease		
	Chronic obstructive pulmonary disease		
	Lung cancer		
	Stroke		
Incidence of smoking-related diseases	Myocardial infarction		32 32 29, 30 31 32
	Heart disease		
	Chronic obstructive pulmonary disease		
	Lung cancer		
	Stroke		
Risk ratio of developing smoking-related diseases	Lung cancer Smokers Former smokers Myocardial infarction Smokers Former smokers Chronic obstructive pulmonary disease Smokers Former smokers Heart disease Smokers Former smokers Stroke Smokers Former smokers	*Male* 8.78/ *Female* 7.48 *Male* 3.01/ *Female* 2.82 4.21 1.47 3.51 2.35 4.22 1.51 2.58 1.34	33 33 35 35 34 34 35 35 35 35
Smoking cessation At first cycle	Combined program Health advice alone	0.24375 0.11875	16 16
After the first cycle	Combined program Health advice alone	0.02 0.02	25
Relapse After the first cycle	Combined program Health advice alone	0.333 0.5264	16 16
After the second cycle	Combined program Health advice alone	0.05 0.05	24 24
Number of attempts at smoking cessation		2	26
Utilities	Smoker with no comorbidities Former smoker with no comorbidities Smoking-related diseases	0.75 0.78 Calculated for different age ranges from	39 39 37, 38
Days of sick leave due to smoking -related diseases	Days of sick leave in smokers (per year) Days of sick in former smokers (per year)	11 7	4
Percentage in employment	Men 16–24 years 25–34 years 35–44 years 45–54 years ≥ 55 years Women 16–24 years 25–34 years 35–44 years 45–54 years ≥ 55 years	41.06% 91.63% 94.05% 90.41% 28.01% 37.23% 86.64% 84.38% 75.65% 18.07%	50 50
Hourly earnings	Men Women	€15.90 €13.60	50
Monthly agreed working hours by type of contract	Men Women	155 h 155 h	50

We assumed an annual rate of relapse of 10% from the first year [24], based on a meta-analysis of clinical trials and prospective studies, and an annual rate of smoking cessation of 2% [25], regardless of the therapy used, age and gender, from the second cessation attempt onwards, this figure also having been used in previous studies [18]. We also assumed that each patient could make two attempts at quitting smoking each year, in line with data from the Spanish National Health Survey [26] (Table 1).

In the absence of valid data from the Spanish population on mortality rates by age and smoking habits, we used figures for the British population by age and smoking habits (smoker, former smoker), calculated in 1994 by Doll et al. [27]. However, before their inclusion in the model, we calibrated these rates seeking to reproduce mortality rates in men and women in the Spanish population in 2018, by applying a corrective factor; the same to smokers and former smoker, which have been subsequently corroborated with the mortality rates provided by the National Institute of Statistics for the year 2018. This procedure has been used by other groups of researchers in cost-effectiveness studies published in recent years [18, 28].

To calculate the number of smokers and former smokers who might develop a smoking-related disease in each cycle, we multiplied the number of smokers/former smokers in each cycle by the incidence of each disease by age and gender (when such disaggregated data were available) (Table 1) [29–32] and the excess risk of developing each disease in the case of smokers and former smokers (Table 1) [33–35].

$$\begin{aligned} {\text{Disease incidence}} = \, & \left( {{\text{Disease incidence in former smokers}}*{\text{Number of former smokers}}} \right) \, \\ + \left( {{\text{Disease incidence in smokers}}*{\text{Number of smokers}}} \right) \\ \end{aligned}$$

Similarly, we calculated the prevalence of each disease as a function of smoking status:

$$\begin{aligned} {\text{Disease prevalence}} = \, & \left( {{\text{Disease prevalence in smokers}}*{\text{Prevalence of smokers}}} \right) \\ + \left( {{\text{Disease prevalence in former smokers}}*{\text{Prevalence of former smokers}}} \right) \\ \end{aligned}$$

The data on prevalence (Table 1) of the different diseases considered were taken from the study by Flack et al. on interventions for smoking cessation [19]. According to this study, the prevalence rates increased with age and differed as a function of gender.

### Health benefits

The values for health-related quality of life (Table 1) of the five smoking-related diseases [36] in healthy population and the corresponding decrease associated with myocardial infarction, stroke, chronic obstructive pulmonary disease, and heart disease were taken from the 2011–2012 Spanish National Health Survey, disaggregated by age and sex, and assessed using the EuroQol 5D-5L [37]. In the case of lung cancer, the decrease of health-related quality of life was assessed using the results of Trippoli et al. [38]. Further, the data on the quality of life of smokers and former smokers with no comorbidities were obtained from a study by Tillmann et al. conducted in 1997 [39]. As in previous studies, when patients had more than one comorbidity, we applied the lowest utility value [18].

### Costs

In the cost analysis from a healthcare perspective**,** we only include direct healthcare costs related to the intervention administered (cost of the text messaging, cost of the messages sent, and costs associated with the visits to health professionals) and related to the smoking related diseases-SRD) [40]. All the costs (Table 2) are expressed in euro for 2018, corresponding inflation rates being applied for each year.

**Table 2:** Costs included in the model

	Combined program	Health advice
Costs of each option/patient	€187.90	€166.95
Cost of general practitioner appointment (2018 portfolio of services of the Basque Health Service)	€58 × 4	€58 × 4
Cost of nurse appointment (2018 portfolio of services of the Basque Health Service)	€24 × 1	€24 × 1
Cost of nurse phone consultation (2018 portfolio of services of the Basque Health Service)	€12 × 4	€12 × 4
Cost of the text messaging program	€17,385.27^a^	
Cost of the messages sent	€3,127.85^a^	
Cost of two CO monitors and mouthpiece	€1,891.5^b^	
Program logo	€431.5^a^	
Annual costs of the treatments of smoking-related diseases		
Incidence-related costs		
Lung cancer	€13,206	44
Stroke	€5,759.50	44
Myocardial infarction	€12,987	43
Chronic obstructive pulmonary disease	€1,672	42
Heart disease	€8,578	43
Prevalence-related costs		
Lung cancer	€13,206	44
Stroke	€3,596.60	44
Myocardial infarction	€3,046	43
Chronic obstructive pulmonary disease	€1,672	42
Heart disease	€685	43
Training costs for the combined program	€1,900	

^a^For calculating the costs per patient, the total amount for each item was divided by the total number of patients in each group (1000)

^b^For calculating the costs per patient, the total amount for each item was divided by the total number of people in both groups

Regarding the analysis from the social perspective, we assumed the direct healthcare costs specified in the previous paragraph and also losses of productivity due to SRD (Table 2) [41–46]. We estimated the disease-related loss of productivity as the reduction in productivity of a worker who is ill or unable to work. Further, for calculating the loss of productivity due to sick leave, we considered the percentage of male and female smokers and former smokers in work, the hourly earnings for men and women, and the mean number of monthly agreed working hours for men and women, all these data being obtained from the Spanish National Statistics Institute [47].

In order to estimate indirect costs and transform them into monetary units, we used the human capital approach [48–50]. This approach converts life years into monetary equivalents considering the mean gross income of each worker. The method is based on the hypothesis that the value of the lost production is equivalent to the wage associated with obtaining the aforementioned production. That is, a day off work represents a loss of production equal to the wage for that same day worked [51]. With this methodology, a single wage, often the mean or the minimum, is applied to all analyzed people. Interestingly, a study published by Suarez-Bonel et al. [4], demonstrated that smokers and non-smokers were on sick leave for an average of 11 days and 7 days a year, respectively. All the parameters entered into the model are listed in Table 1.

### Model validation

The model was validated internally and externally. For the former, we followed all the recommendations of Halpern et al. [51] and Nuijten et al. [52]. In addition, according to McCabe et al. [53], the results of the model can only be properly validated in one way, that is, by comparing the modelled estimates with the values obtained in real life, which could be called predictive validity. To address this, we calculated the life expectancy of men and women at different ages based on our model and compared it with the figures provided by the Spanish National Statistics Institute (real data). For the external validation, we used the LYGs thanks to smoking cessation at different ages and the life years lost due to smoking at 40 years of age, comparing the results with those of Ozasa et al. [54]. In this particular case we considered a utility of 1 and a discount of 0.

### Deterministic sensitivity analysis

We performed a univariate deterministic sensitivity analysis to assess the change in ICUR as a function of the changes in the effectiveness values of the combined program or the motivational advice alone, and the age of starting program (50 years old). We have also carried out a multivariate sensitivity analysis modifying both probabilities of smoking cessation at the same time, and also equalizing the probability of relapse at the first cycle (0.5264) in the both alternatives.

The effectiveness values for the combined program used to perform the sensitivity analysis lie within the 95% confidence Interval (17.71–31%) as did the corresponding values for treatment effectiveness of motivational advice alone (6.86–16.88%) [16].

We have also repeated the sensitivity analysis with a 6% discount rate.

### Threshold

The reference threshold that has been employed to consider or our combined program, as cost-effective is the estimated by Vallejo-Torres et al. [55] in 2018, for the Spanish NHS (€22,000).

The cut-off point of smoking cessation probabilities o *NOT cost-effectiveness* identified for women when the probabilities of relapse are equalized are 0.177 and 0.162 for combined program and health advice respectively.

## Results

The increase in costs at short-term (at 6 months-moment of the end of program), associated with mobile phone messaging for a cohort of 1000 smoking people was €22,850 from a healthcare perspective. At the end of the program there was also a bigger percentage of people quitting smoking in the combined program group (244 versus 119), which translates to an additional cost of €183 per tobacco quitter, at this moment.

From a healthcare perspective, the increase in costs through the entire life of participants was €7.4 and €1,327 per QALY gained (ICUR) for men and women, respectively. From the social perspective, the alternative treatment was dominant, with savings of €5,398 and €3,290 per QALY gained (ICUR) for men and women respectively (Table 3).

**Table 3:** Results of the cost-effectiveness analysis. Base case (cost and QALYs increase for a hypothetical cohort of 1000 patients)

Healthcare perspective							Societal perspective						
Combined program		Health advice alone		Difference between the options			Combined program		Health advice alone		Difference between the options		
Mean costs (€)	Mean QALYs	Mean costs (€)	Mean QALYs	ΔCost (€)	Δ QALYs	ICUR €/QALY	Mean costs (€)	Mean QALYs	Mean costs (€)	Mean QALYs	ΔCost (€)	Δ QALYs	ICUR €/QALY
Men													
2,576	20.61	2,565.80	20.59	199.93*	27.07*	7.40	22,014	20.61	22,160	20.59	− 146,130.25*	27.07*	− 5,398 (DOMINANT)
Women													
1,888	20.98	1,855	20.96	33,333*	25.10*	1,327	16,829	20.98	16,912	20.96	− 82,632.81*	25.12*	− 3,290 (DOMINANT)

A deterministic sensitivity analysis was performed to assess whether the results were maintained when certain variables were modified. Table 4 shows that as the difference in the probability of quitting smoking between the combined program and health advice alone increases, both QALYs gained and ICUR increase. Table 5 shows that increasing the age of smoking cessation, more saving are generated for the system. Table 6 shows that equalizing the probability of relapse at the first cycle in both alternatives (0.5264) from healthcare perspective, the combined program is not cost effective (€ 48,998 cost/Qaly) for the following probabilities of quitting tobacco (0.177 CP and 0.169 HA) in women. From society's perspective the model is always dominant in men. In women it is dominant when the probability of relapse is unchanged. When the probabilities of relapse are the same in both alternatives (0.5264) the model is cost effective for the following probabilities of quitting tobacco (0.177 CP and 0.119 HA) and it is not cost effective for the following probabilities of quitting tobacco (0.177 CP and 0.169 HA).

**Table 4:** Results of the cost-effectiveness analysis. Univariate sensitivity analysis. Changing the probability of smoking cessation

Assumption modified	Combined programme						Health advice alone					
Probability of smoking cessation	0.1772^a^		0.24375^a^		0.3103^a^		0.0686^b^		0.11875^b^		0.1688^b^	

	ICUR (€/QALYs)											
	Men	Women	Men	Women	Men	Women	Men	Women	Men	Women	Men	Women
Healthcare perspective	115	2,426	7	1,327	− 36	888	− 20	1,046	7	1,327	51	1,782
Societal perspective	− 5,312	− 2,245	− 5,398	− 3,290	− 5,432	− 3,707	− 5,409	− 3,548	− 5,398	− 3,290	− 5,404	− 2,281

^a^The probability of smoking cessation with health advice alone remains constant (0.11875)

^b^The probability of smoking cessation with combined program remains constant (0.34275)

**Table 5:** Results of the cost-effectiveness analysis. Univariate sensitivity analysis. Changing the age of smoking cessation (50 years old)

	ICUR (€/QALYs)	
	Healthcare perspective	Societal perspective
Men	− 2,785	− 7,591.81
Women	− 796.27	− 4,351.31

**Table 6:** Results of the cost-effectiveness analysis. Multivariate analysis

Healthcare perspective ICUR (€/QALYs)													
		Probability of smoking cessation with combined program											
		Without modifying the probabilities of relapse						The same probabilities of relapse in both alternatives					
		0.069		0.119		0.169		0.069		0.119		0.169	
		Hombre	Mujer	Hombre	Mujer	Hombre	Mujer	Hombre	Mujer	Hombre	Mujer	Hombre	Mujer
***Probability of smoking cessation with HEALTH ADVICE***	0.177	42.660	1,672.04	115.42	2,426	289.430	4,260.40	149.17	2,747.60	392.43	5,309.79	3,553.06	48,998
	0.244	− 19.69	1,046.04	7.4	1,327	51.125	1,782.21	40.04	1,640.11	110	2,362.79	273.44	4,079.09
	0.310	− 50.09	742.66	− 36.140	888.28	− 16.674	1,091.94	− 8.99	1,147.87	23.82	1,486.36	79.84	2,067.81

On the other hand, when repeating the sensitivity analysis with a 6% discount rate, we have obtained very similar results.

### Model validation

The life expectancy estimated from our model is very similar to the data provided by the Spanish National Statistics Institute for different ages (Table 7). The results of the external validation are shown in Tables 8 and 9. Smoking cessation at an age ≤ 40 or < 50 years old translates to 4.2 and 3.9 LYGs, respectively, being these results similar to those of Ozasa et al. [54]. At older ages, the differences between Ozasa et al. and our group increase slightly. In addition, the life expectancy values calculated from our model and from that of Ozasa et al. [54] as a function of smoking status and gender [54] at the age of 40 years old are very similar, finding the largest difference in the case of smoking men (2.7 years) whereas the smallest difference in non-smoking men (0.1 years).

**Table 7:** Internal validation of the model with the life expectancy for the Spanish population

	Patient life expectancy for 2018 (data from the Spanish National Statistics Institute)	Model
Men		
16 years	64.77	64.77
30 years	51.03	51.7
50 years	31.86	31.02
Women		
16 years	70.11	69.6
30 years	56.24	56.34
50 years	36.76	37.4

**Table 8:** External validation.Years of life gained after smoking cessation at different ages

Sex	Age at smoking cessation (years)	Years of life gained	
		according to our model (years)	according to Ozasa et al.* (years)
Male	40	4.2	4.8
	50	3.9	3.9
	60	3.3	1.6

**Table 9:** External validation. Life expectancy as a function of smoking status at 40 years old

Smoking status	Age	Men		Women	
		Our model	Ozasa et al.	Our model	Ozasa et al.
Former smokers	40 years	40.03	40.8	44.2	42.4
Smokers		35.8	38.5	39.8	42.1
Non smokers		42.3	42.4	46.5	46.1

## Discussion

This economic assessment shows that the use of text messaging as a tool to support health advice is cost-effective from a health care perspective, given that it leads to health benefits and reduces costs. From the healthcare perspective, the ICUR is far below the threshold of €22,000 calculated for the Spanish health system [55]. The ICUR regarding the use of the combined program for smoking cessation compared to usual practice represents an increase in costs of €1,327 and €7.4 for each QALY gained for women and men, respectively.

Considering a social perspective, the combined program is an alternative that results in savings of €5,398 and €3,290 per QALY gained for men and women respectively. These saving costs are related to the fact that former smokers have less risk of suffering from SRD. This entails fewer work leaves, thus generates savings costs from society perspective.

It is more cost-effective in men as they are at greater risk of developing disorders related to smoking than women, and proportionally, the benefits of smoking cessation translate to a greater reduction in the risk of developing common smoking-related diseases in men. These benefits of the program are maintained when we modify the assumptions in the different sensitivity analyses carried out.

The design selected in the present study aimed to maximize the validity of the results. In particular, a Markov model was chosen as the nature of the process under study is chronic with health states changing over time and associated with events due to risk exposure [23]. The recommendations of Halpern et al. [51] and Nuijten et al. [52] for selecting the data to input to the model in terms of costs, effectiveness and probability of smoking were followed. In addition, effectiveness data was selected from a clinical trial carried out by our research team [16]. Last but not least, data on costs for smoking-related diseases for the Spanish population, when available, and utility data for these diseases were obtained from the Spanish population.

Nonetheless, there are several limitations when interpreting the results of this study. First, the mortality rates were taken from the data of Doll et al. [27] for the British population corrected for smoking status, as we did not have access to adjusted rates for the Spanish population. On the other hand, we calibrated these rates to reproduce the mortality rates for men and women in the Spanish population, assuming a risk that is proportional to the baseline risk for former smokers and smokers. Second, as with previous economic assessments, this study may potentially underestimate the benefits of reinforcement through text messaging as a tool to support health advice for smoking cessation, since it does not assume the effects of passive smoking reduction or other less common smoking-related diseases [15]. As a consequence, the study may also underestimate the potential savings associated with the intervention, as it does not take into account the costs of treatment of these smoking-related health problems. Third, our study was based on mean costs of the diseases most commonly associated with smoking, these figures varying with disease severity. Fourth, due to the lack of valid data on incidence of smoking related diseases (SRD) for Spanish smoker population, data on incidence of SRD come from different countries, but at least, all data on Incidence come from European Community Countries. Despite these limitations, results from the present study come along with those reported by others: 0.5 QALYs for former smokers [18], 0.069 QALYs for former smokers [56] and 0.10 QALYs for former smokers [57]. These results are also consistent with previous economic assessments showing that smoking cessation interventions using mobile phones are cost saving [18, 58] and cost effective [59].

Interestingly, the program studied herein becomes more cost effective as we increase the age at initiation of the intervention, given that it increases the probability of developing a smoking-related disease and the benefit of smoking cessation is greater, as found by Guerriero et al. [18] with larger savings the older the age of the study subgroup. The numbers of YLG related to smoking cessation obtained in our model are very similar to those found by Ozasa et al. [54], at the ages of 40 and 50 years old, with the difference being greater above 60 years of age. A potential explanation for this difference is that the non-smokers from the Ozasa cohort [54] were less healthy, that is, they may have had health problems that made them less likely to smoke, and hence, the number of YLG as a result of smoking cessation was smaller in this older age group.

The WHO Framework Convention on Tobacco Control (FCTC) [60] proposed a series of measures for the prevention and control of non-communicable disease. With the combined program our intention is to reinforce the measure (O) Offer-ofrecer, to help to quit tobacco use. The 2018 International Conference on Tobacco Control, held in Madrid, 14–16 June 2018, concluded that the measures that should be adopted by public authorities in Spain with regards to Article 14 of the FCTC (Demand reduction measures concerning tobacco dependence and cessation) include facilitating access by smokers to health professionals trained in managing smoking treatments and fund clinical, behavioral and pharmacological interventions proven to be effective and safe in the treatment of smoking.

## Conclusions

The present study clearly shows that the use of motivational messaging as a tool to support health advice provided by primary health care professionals is a cost-effective strategy from the healthcare perspective, and a dominant strategy from the societal perspective, and hence, following the recommendations of this aforementioned conference, such a strategy should be adopted. Notably, the National Institute for Health and Care Excellence has recently included the use of text messaging as an effective tool for smoking cessation in its recommendations [61].

The potential transfer of this program to primary care clinical practice is feasible given the low associated costs. It is estimated that at least 70% of the population seek medical attention through their general practitioner at least once a year, and smokers do so more often than non-smokers. Thus, primary care provides a great opportunity to introduce and promote our program [62].

## Data Availability

Material is available in Bioaraba health research institute for any request of scientific community.

## Abbreviations

ICUR: The incremental cost-utility ratio
LYGs: Life years gained
QALYs: Quality-adjusted life years
SRD: Smoking related diseases
FCTC: WHO Framework Convention on Tobacco Control
